# How Social Experience and Environment Impacts Behavioural Plasticity in *Drosophila*

**DOI:** 10.1080/19336934.2021.1989248

**Published:** 2021-12-02

**Authors:** Molly Chen, Marla B. Sokolowski

**Affiliations:** a Department of Ecology and Evolutionary Biology, University of Toronto, Ontario, Canada; b Current Affiliation: Department of Biology, University of Waterloo, Waterloo, Ontario N2L 3G1 Canada; c Child and Brain Development Program, Canadian Institute for Advanced Research (CIFAR), Toronto, Ontario M5G 1Z8, Canada

**Keywords:** Social environment, *Drosophila*, behaviour, plasticity, development, ecology, evolution, model organism

## Abstract

An organism’s behaviour is influenced by its social environment. Experiences such as social isolation or crowding may have profound short or long-term effects on an individual’s behaviour. The composition of the social environment also depends on the genetics and previous experiences of the individuals present, leading to additional potential outcomes from each social interaction. In this article, we review selected literature related to the social environment of the model organism *Drosophila melanogaster*, and how *Drosophila* respond to variation in their social experiences throughout their lifetimes. We focus on the effects of social environment on behavioural phenotypes such as courtship, aggression, and group dynamics, as well as other phenotypes such as development and physiology. The consequences of phenotypic plasticity due to social environment are discussed with respect to the ecology and evolution of *Drosophila*. We also relate these studies to laboratory research practices involving *Drosophila* and other animals.

## 1. Introduction

A social environment can be defined as the composition of individuals of the same species surrounding the individual of interest. Social environments can interact directly or indirectly on the individual of interest [1]. Variables in the social environment can include the number of other individuals and their behavioural phenotypes which can be influenced by genotype, age, past experiences, and abiotic and biotic envrionmental variables. Together, these factors lead to differences in the quantity and type of social interactions that an individual experiences throughout their lifetime.

The ability for social interactions to impact an organism’s behaviour, fitness, and physiology have been well observed in many species. Laboratory and field experiments have traditionally focused on ‘social animals’ such as mammals and birds [2,3]. Research in human biology and sociology has also revealed important influences that the social environment has on issues from epidemiology to economics [4,5].

One of the most well-known model organisms of the last century is the fruit fly *Drosophila melanogaster*. Discoveries about the genome of the fly paved the way to understanding patterns of inheritance, development, and behaviour [6]. Despite being a non-eusocial insect, the life history and natural habitat of *Drosophila melanogaster* is highly dynamic with respect to their social environment [7]. As larvae, they forage in rotting fruit, surrounded by other larvae. Adults aggregate on food sources to compete for mates and oviposition sites. Social behaviour, defined here as interactions between two or more individuals of the same species, has been well studied in *D. melanogaster*, with an emphasis on linking genes to behavioural phenotypes such as courtship and aggression [8]. More recently, research has expanded to behaviours of groups of flies, the neural underpinnings of these behaviours and the emergent properties of the groups [e.g. 9, 10], leading to interest in how *Drosophila* respond to their surrounding social environment.

**Figure 1.**
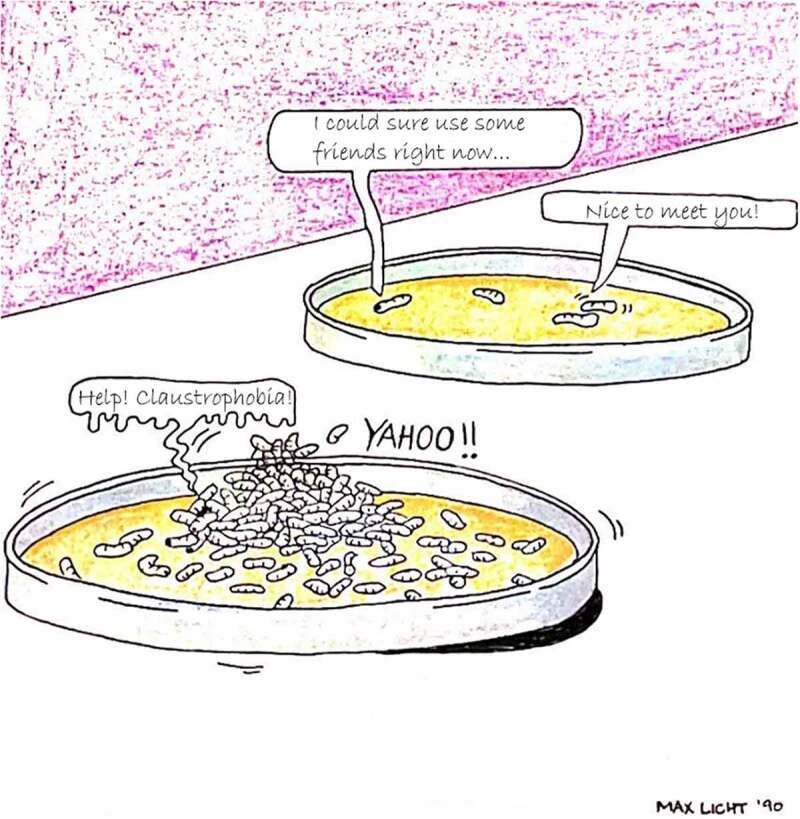
*Drosophila* may be exposed to various social environments at different life stages. For example, during the larval stage, conditions such as social isolation or crowding may lead to both short and long-term effects on an individual’s behaviour, development, and fitness. Figure modified from Figure 3 in [18]. Illustration by Max Licht

In the present review, we focus on experimental findings related to the social behaviour and the social environment of *D. melanogaster* (although other species of *Drosophila* are also discussed for comparison). We examine several aspects of the social environment that have been manipulated in the literature: isolation, crowding, and the composition of the social environment (an example is illustrated in Figure 1). There is a diverse range of effects of these variable social environments on behaviour, development, and physiology. We discuss the findings from the perspectives of the ecology, evolution, neurobiology, and genetics of *Drosophila*, as well as their implications for other animals (e.g. mammals). This article is not intended to be a comprehensive review of all research on *D. melanogaster* social behaviour and the social environment, but rather a selection of literature chosen to illustrate key themes and examples of interest.

## 2. Part 1: social isolation

Social isolation can be defined as the lack of contact and social interactions between an individual and members of its own species. In humans, prolonged isolation is currently a growing problem in the older adult population which is associated with its many detrimental effects on health and well-being, making it an important area of study [11,12].

Experimental studies of the effects of social isolation have historically focused on mammalian subjects such as rats and primates, due to their findings being more applicable to human psychiatric and neurological conditions [13–16]. However, differing levels of isolation can be experienced by a variety of species, including those not typically considered to be social animals (animals in which parental care, group living, and/or cooperation are part of their life history). Bailey and Moore 2018 quantify social isolation in this context as the difference between the optimal amount of social interaction for an individual and the actual amount experienced [17]. In their review [17], they describe how social isolation can alter evolutionary dynamics of populations, using examples from species such as the field cricket *Gryllus pennsylvanicus* showing how isolation impacts sexual selection [19], and from the invasive cane toad *Rhinella marina* displaying behavioural plasticity depending on the degree of isolation in the population [20].

In this section, we review experimental evidence for the impact of social isolation on *Drosophila*, and how these findings inform our understanding of their behaviour in nature and in the laboratory.

### 2.1. Courtship, mating, and evolution

Studies on various *Drosophila* species have shown that even closely related species can have profoundly differing responses to social isolation. One example is in courtship and mating success. *D. silvestris* show decreased mating success when individuals were reared in isolation [21]. Isolated *D. silvestris* individuals often exhibit ‘escape’ behaviour when presented with a mating partner. Isolated males were also unable to court effectively, and isolated females frequently interrupted courtship attempts. In contrast, socially isolated *D. paulistorum* males have increased courtship behaviour and were more successful in competing for mates [22]. The finding that these two species react in different directions to the same experience (social isolation) may contribute to our understanding of the forces shaping the speciation and sexual isolation of drosophilid species and subspecies based on mating behaviour.

Experiences and behaviour early in life can affect the reproductive fitness of *Drosophila* in adulthood. In species where courtship behaviour is learned, individuals may require and even seek out social interactions earlier in life to develop successful mating behaviour. For example, *D. melanogaster* males refine their courtship behaviour based on past experiences with mated and unmated females; experienced males begin courting mated females more slowly and unmated females more quickly compared to inexperienced males [23]. This phenomenon requires males to form associative memories with the signals communicated by females during previous courtship encounters. A common assay for testing learning and memory in *Drosophila* males is known as courtship conditioning, where an inexperienced male is paired with a mated unreceptive female and subsequently the male fly decreases courtship behaviours for several hours towards other females [24]. Other species such as the aforementioned *D. paulistorum* may benefit from social isolation. Kim & Ehrman 1998 [22] discuss the possibility that social isolation may lead to greater access to nutritional resources resulting in socially isolated males being larger and therefore more successful in mating.

Mechanisms of reproductive behaviour in *Drosophila melanogaster* have historically been studied using genetic mutants. The neurogenetics of sensory systems and gene functions in the central nervous system (CNS) related to courtship and mating have been extensively studied [reviewed in 25]. The first mutation identified that alters male courtship song was in the X-chromosomal gene cacophony (*cac*), which interrupts the typical song pattern produced by males [26]. More recently, the social experience of male flies has also been studied for its role in courtship behaviour. Marie-Orleach et al. [27] found that *D. melanogaster* males in the presence of con- and heterospecific males produce courtship songs of longer duration than isolated males. This effect may be due to the social interactions helping to trigger reproductive maturity, modulated by signalling with CREB-binding protein (CBP) to enhance the efficacy of juvenile hormone (JH) [28]. Mutant male flies with lower levels of JH synthesis show reduced courtship behaviour [29].

Same-sex courtship behaviour (SSB) has also been observed in males and is influenced by prior experience with both males and females [22,30]. SSB can contribute to reproductive fitness in males [31]. Younger males receive a fitness benefit as they gain experience through SSB, which leads to more success in courting females later in life. Older males that already have mating experience do not benefit and avoid courtship with younger males, further supporting the ideas that the life stage and timescales of the social environment are important in determining behavioural plasticity in courtship behaviour.

The above-mentioned studies on social isolation in *Drosophila* males reveal the importance of social interactions in the plasticity of courtship behaviour. Males can respond to social interactions in a complex manner, by integrating past experiences with other males and current perceptions of mate competition. Subsequent research could investigate the effects of social isolation on female behaviour, as mate choice is a plastic phenotype [32].

### 2.2. Development in larvae and adults

The timing of social isolation is important in determining how it affects individuals. In *Drosophila*, early larval stages are particularly sensitive to periods of isolation [33]. Foraging larvae generally form aggregated distributions based on visual attraction to other larvae. Slepian et al. [33] showed that when larvae are socially isolated during the first larval instar (L1 stage) they do not form aggregations, suggesting that this behaviour is learned during an early critical period in development; isolated larvae are unable to recognize the movement patterns of other larvae and are no longer attracted to them. Early isolation can therefore impact the social behaviours of older larvae (for example, cooperative foraging performed by third instar larvae, which will be discussed later in this review).

Foraging behaviours influence nutrient acquisition, which is crucial during the larval stage in order to reach the critical mass required to pupate [34]. Therefore, social interactions among *Drosophila* larvae and aggregation during foraging may be an evolutionary adaptation that allows for higher efficiency in nutrient uptake when feeding in groups. Further research is required to determine the effects of social isolation on larval nutrition. Experiments should be performed comparing isolated to group reared larvae, when given adequate amounts of food, to determine whether there are differences in feeding rate and nutrient absorption. Developmental milestones such as the time required to reach critical mass/pupation and the weight of the larvae at each stage could also be measured as well as adult fitness.

Unlike larvae, adult flies can disperse over long distances and therefore may be more likely to experience prolonged periods of social isolation [35]. Leech et al. [36] studied the effects of isolation on health and ageing throughout the lifespan of adult *D. melanogaster*. They found that isolating adults extends their lifespan compared to rearing adults in same-sex pairs. However, different sex-specific effects for the immune response (wounding) and functional senescence (climbing ability) were found in isolated vs. paired adults. For example, isolated females declined in climbing ability as they age faster than paired females, while the reverse was true for isolated males compared to paired males. Researchers hypothesized that sexually dimorphic responses to isolation are due to the increased cost of mating for males. Paired males anticipating mate competition may elicit costly physiological responses, such as increased sperm production, even if the sperm is not used [36]. In a follow-up study, [37] showed that immune responses were also linked to social environment, age, and sex. Specifically, social interactions in females increased lifespan post-infection and reduced the expression of stress response genes (*Turandot* (*Tot*) genes, which encodes a humoral protein that increases resistance to high temperature). These studies suggest that social contact between females is overall less stressful than between males.

The studies discussed so far have focused on the effects of isolation on either the larval stage or the adult stage. Further investigation is needed to determine how the larval social environment may have long-term effects on behaviour and health in adulthood. Early adversity experienced by larvae in terms of food deprivation is known to impact adult exploratory behaviour in a novel environment [38], which is reflective of our understanding of mammalian development. In rats, macronutrient deficiencies lead to reduced performance of individuals in learning and memory related tasks [39]. Protein, iron, and iodine deficiencies in human children can permanently impair cognitive function [40]. One study demonstrated that social isolation in *D. melanogaster* larvae resulted in more fragmented sleep patterns in wild-type adult flies, with females being more affected than males [41]. Additional research could explore whether larval social experiences affect phenotypes such as adult mating, aggression, and foraging.

### 2.3. Sleep and neurophysiology

There exists a plethora of research on the negative effects of sleep deprivation in humans, from physiological [42] to social [43]. As a result, researchers are interested in the factors that lead to altered sleep patterns and sleep requirements.

Social isolation impacts sleep patterns in mammals. In mice, social isolation acts as an additional stressor with sleep deprivation, with paired mice recovering from sleep deprivation more effectively than isolated mice [44]. Sleep quality in humans is inversely associated with social isolation and loneliness in older adults [45].

Interestingly, similar effects of isolation on sleep exist in *D. melanogaster*. Isolated flies were found to sleep less during the day than socially enriched flies and this effect was proportional to the size of the group [46]. Effects of isolation also persist across life stages, as mentioned in the previous section [41]. Furthermore, isolation-induced sleep deprivation in both flies and mice has been shown to induce cellular stress and the unfolded protein response (a response to the build-up of misfolded proteins in the endoplasmic reticulum), which is hypothesized to contribute to the negative health outcomes of social isolation [47]. A recent study also linked chronic social isolation in flies with both reduced sleep and an altered brain state that signals starvation, using behavioural and transcriptome analyses [48]. The researchers connected the behavioural changes in chronically isolated flies (sleep loss and overconsumption of food) to potential consequences on sleep and food-related behaviours in humans that suffer from chronic loneliness [48].

A few studies have examined the link between isolation-induced behavioural changes and the physiological mechanisms involved. Liu et al. [49] tracked social network parameters (a measure of behaviour) alongside gene expression changes in response to social isolation. The researchers found that the expression of metabolic genes changed within just one day (causing an increase in lipid content in flies), whereas behaviour [e.g. locomotor activity, number of social interactions) changed across six days. Agrawal et al. 2019. examined the epigenetic effects of social isolation vs. social enrichment on sleep behaviour [50]. By identifying changes in histone modification marks and mRNA expression levels in dopaminergic neurons, they found that some genes encoding activity-related transcription factors (ARG-TFs] respond to social environment changes. Epigenetic modifications occurred within just 4 days of social isolation. Knockdowns of these ARG-TFs rescued the social effects on daytime sleep.

Much is known about how isolation adversely impacts social creatures and their health. The studies we have discussed on isolation’s effects on sleep and other physiological responses in *Drosophila* show that these effects could be conserved in simpler model organisms, suggesting that the benefit of social interactions evolved earlier than previously thought.

### 2.4. Aggression, genetics, and gene expression

Isolating adult flies for several days changes their behaviour towards other flies. They show a lower social space measurement (SSI, or distance between two flies) compared to non-isolated counterparts [51]. This behaviour is modulated by dopaminergic signalling [52].

Aggressive behaviours also increase in both sexes after social isolation. Females show increased aggression over food sources [53], and male territorial aggression [54]. In *D. suzukii* males display more aggression than socially reared males, and socially reared females lack aggressive behaviours [55].

Laboratory studies in aggression are important in forming hypotheses for how plasticity in aggressive behaviours evolved. For example, isolation could be highly correlated ecologically with food scarcity and other adverse conditions, leading to increased aggression to maximize competitive ability. Natural populations of *Drosophila* face many additional challenges compared to laboratory populations, which could lead to periods of isolation for individuals. Flies are exposed to many pathogens, parasitoids, predators, and fluctuating abiotic conditions (especially in temperate climates where overwintering is required) and reviewed in [7,56]. Isolation may also serve to alert individuals of these conditions, and aggression may be one of many responses to prepare for adversity.

More recently, research has identified genes that mediate aggressive behaviours and have demonstrated that their expression is influenced by social isolation. Ueda & Wu 2009 followed up on an earlier study [53] by examining larval social isolation; larvae that were reared in isolation from the time of hatching to the late third instar phase had increased neuromuscular junction transmission and nerve/muscle excitability [57]. They identified two genes, *Hyperkinetic* (*Hk*) and *glutathione S-transferase-S1* (*gsts1*) that were necessary for both plasticity in adult aggression and larval neuromuscular excitability. *Hk* and *gsts1* mutants did not show differences in aggression and nerve/muscle excitability when comparing isolated and group reared individuals. *Hk* and *gsts1* are involved in redox regulation and the Reactive Oxygen Species (ROS) stress response, suggesting that ROS regulation mechanisms can influence synaptic functions at neuromuscular junctions, which may lead to behavioural changes.

Agrawal et al. 2020 [58] found that the neuropeptide Drosulfakinin (*Dsk*) is downregulated in adult flies in response to 4 days of social isolation. In this case, the connection to behaviour was clear, as knockdown and genetic silencing of *Dsk* also led to increased aggression. *Dsk* is a homolog to mammalian cholecystokinin (CCK), which functions both as a signal hormone in the gut and neuropeptide in the central nervous system. Elevated CCK levels in humans have been linked to increased anxiety [59] and hallucinatory disorders [60].

A genome-wide analysis was done on male flies in a socially isolated compared to a socially enriched environment to examine genetic and environmental contributions to differences in aggression [61]. This study found genetic variation that contributes to aggressive behaviour, as well as significant genotype-by-environment interactions. Many of the identified genes are associated with aggression in mice, and have homologs in humans associated with psychiatric disorders.

This section has discussed the study of genetics and gene expression mediating aggression in *Drosophila*, and the potential for this research to provide more information on how social environments influence homologous genes in humans associated with social behaviour and psychiatric conditions. Future research in this area could examine mutations of these genes in *Drosophila* as well.

## 3. Part 2: crowding and density

On the opposite end of the spectrum to social isolation, high population densities represent another challenge for *Drosophila* in both natural and laboratory populations [62]. In the wild, young larvae are confined to the piece of rotting fruit that they hatched on and are susceptible to competition from other larvae. Husbandry of lab populations must also take care to avoid crowding of flies in their confined environments, as they will reproduce rapidly given the right conditions. Crowding of larvae can be detrimental to the fitness of the resulting adults, in terms of body size and fecundity, which we will discuss in this section.

The effects of crowding are also seen in human societies. Management of dwindling natural resources becomes more difficult, environments become polluted and deteriorated, and social problems arise (such as the availability of schooling and employment) [63]. Studying the effects of crowding on animals also impacts our decisions on care protocols for domestic and laboratory animals, since it is important to understand how to mitigate crowding-related stress and the spread of infectious diseases [64].

This section reviews what is currently known about the effects of high population densities on *Drosophila*, with emphasis on the behavioural adaptations that flies have evolved to succeed in highly crowded environments.

### 3.1. Adult behaviour, evolution, and ecology

Most research on the effects of crowding in *Drosophila* is focused on larvae. Adults with their higher dispersal ability are assumed to be less affected by crowding than larvae. However, laboratory studies have shown that adults can show behavioural plasticity based on the perceived levels of crowding in their environment. For instance, Rooke et al. [65] determined that flies can sense both group size and density by regulating their movement behaviours and interaction levels accordingly. It was determined that *lush*-expressing cells (required for detection of cis-vaccenyl acetate, a male-specific hormone influencing aggregation in *Drosophila*) [66] were necessary for detecting group size. The ability to fine-tune individual behaviour based on group parameters is important for participating in social networks and group behaviour, which will be discussed more in detail in section 3 of this review.

Reproductive fitness can be a driving factor for the evolution of different mating behaviours based on density. Courtship in *Drosophila* is dependent on many integrated sensory cues, with pheromonal communication via cuticular hydrocarbons being among the most important [67,68]. Both male and female flies release species-specific pheromones that promote aggregation, which support finding a mate. Male courtship behaviours remain consistent across moderate levels of crowding, suggesting that courtship may have evolved under aggregated conditions [69]. However, the crowded environment in this study was relatively short term (males were introduced to environments of varying density and time to first mating was recorded). Interestingly, high-density conditions have been shown to induce plasticity in mating behaviours across life stages and across generations. Crowded larval environments produce adult males with increased courtship behaviour [70]. Adult males that experience sperm competition from being housed with other males produce male offspring that also exhibit longer mating duration [71]. From these studies, it appears that long-term crowding is more predictive of mate competition than short-term crowding, suggesting that natural populations often experience transient phases of highly aggregated individuals, but this is not necessarily reflective of a consistently high overall population density.

A potential consequence of high population density is increased competition for mates. Omesi et al 2021 studied the effects of mate competition on *Drosophila* male behaviour and physiology, from the perspective of sexual deprivation rather than the presence of rival males. Males that experience repeated failures to mate during encounters with females display more aggressive behaviour and form social networks with lower interactions between-group members [72]. Sexually deprived males also increase their production of seminal fluid and copulation time with receptive females, which reduces re-mating rate [72]. Plasticity in mating behaviour under high-density conditions can enhance short-term reproductive fitness by increasing aggression to compete for a mate, and prolonging copulation (mate guarding) [72]. Another experiment on sexual deprivation found that males that recently experienced rejection from a female were more likely to seek out and consume ethanol compared to males that were permitted to mate, which may have implications on the social influences on addiction [73].

Future research in this area could focus on how population density influences dispersal behaviour. Individual differences in *Drosophila* dispersal are dependent on the distribution of food patches across an environment, as well as genetic variation [74]. Perceptions of density may also play a role in dispersal as individuals make decisions based on trade-offs between energy expenditure to find food sources and competition for local food sources. While density-dependent dispersal has been identified and modelled for many species [75–77], there is little research on the mechanisms that animals use to modulate this behaviour. In flies, pheromone signalling could mediate an ‘optimal’ level of aggregation in adults that maximizes the ability to acquire food and reproduce while minimizing competition. In other words, density and dispersal could be indirectly regulated by pheromone sensing in a concentration-dependent manner. Both laboratory and field studies could be used to answer these questions.

### 3.2. Larval crowding, development, and behaviour

Within the larval stage, density is an important factor in determining many food-related behaviours (Figure 1). One behaviour selected for under high-density larval conditions is a higher feeding rate (measured by sclerite retractions per second) [78,79]. Larvae also vary in their foraging pathlength (distance travelled while feeding) and can be classified as ‘rovers’ or ‘sitters’, with rovers having longer pathlengths compared to sitters [80]. This behavioural polymorphism is primarily due to allelic variation in the *foraging* (*for*) gene, which codes for a cGMP-dependent protein kinase [81]. Density-dependent selection over several generations showed that the rover phenotype is selected for at higher density populations [82]. In these studies, researchers hypothesized that selection on the *for* locus may involve differences in the relative fitness associated with each allele under different environmental conditions. Food availability, distribution, and the social environment (density) may vary over space and time. If the different foraging strategies of rover and sitter larvae are more suited to some environments than others, then this could result in changes in allele frequencies in natural populations.

As previously discussed, larvae are visually attracted to the movements of other larvae [33]. Furthermore, they prefer to aggregate on patches of food occupied by other larvae [83]. Potential negative consequences of aggregation include competition from other larvae, as well as an increased risk of parasitic infections and disease. However, it is hypothesized that one major benefit of aggregation is to disrupt the growth of fungal competitors [84], as larvae tend to aggregate more often on patches of food infected with fungal colonies. In a later study, Trienens & Rholfs 2020 found that high densities and larger groups of larvae are more effective at suppressing fungal growth, indicating that there is an optimal density for group fitness [85]. Additionally, variation in *for* contributes to variation in suppressing fungi, with sitters being more effective than rovers [85], suggesting that variation in *for* influences larval aggregation behaviour. This is supported by data showing that adult sitters tend to aggregate more than rovers [86]. Environmental variation in the presence of infectious fungi on *Drosophila* food sources could therefore impose an additional selective force that contributes to the maintenance of this behavioural polymorphism in nature.

Remarkably, high-density conditions promote a novel cooperative phenotype observed in *Drosophila* larvae known as cooperative foraging. Dombrovski et al. 2017 first characterized cooperative foraging in lab populations of wild-type and mutant larvae. When upper layers of food had been consumed and liquified through the release of wastes, larvae orient themselves and coordinate their movements in the same direction while digging into the media to reach deeper layers of fresh food [87]. It is hypothesized that this behaviour allows for more efficient burrowing while maintaining access to oxygen through the creation of a larger cavity that does not collapse. Larvae form these coordinated clusters through visual and mechanosensory cues, as mutants lacking these sensory abilities show much lower cluster frequency [87]. Prior social experience during the early L3 phase is also necessary for larvae to join clusters [87]. Larvae also display kin recognition in the context of cooperative foraging, by increasing the frequency and size of feeding clusters when populations consist of more closely related individuals [88].

Cooperative foraging has been linked to fitness benefits in adults (measured via wing size), at the cost of increased development time [89]. There is much potential for additional research into how cooperation affects larval feeding behaviour and the specific mechanisms in which this increases fitness. Drombrovski et al. 2020 state that the increased development time in larvae under crowded conditions is due to the lower nutrient concentrations present [89]. There could be a few hypotheses for why cooperative foraging leads to increased adult fitness under these conditions. For example, larvae in cooperative clusters could reach patches of nutrient-rich food that individual larvae cannot (with the time required to burrow as a cluster explaining the increased developmental time). Alternatively, larvae in clusters could have differences in feeding rate and absorption that allow them to extract more nutrients from poor-quality food, although it is slower than non-cooperative foraging. Experiments analysing larval growth rate, food uptake, and nutrient absorption in clusters (compared to larvae reared in the same environment that do not form clusters) would help answer these questions.

The above-mentioned studies are important in establishing that larvae can sense their social environment and make decisions based on population density. Larval behaviour can be important in maximizing fitness during the larval stage and reaching maturity, as well as preparing an individual for adulthood under similarly crowded conditions. Furthermore, examples of insect cooperation are rare outside of eusocial insects. One such instance of cooperation is found in the burying beetle *Nicrophorus vespilloides*. In this species, adult beetles care for their offspring by defending their food source (a vertebrate carcass), and often help feed larvae by regurgitating pre-digested food [90]. When parental care is absent, siblings are more likely to cooperatively feed on the carcass as a way of maximizing their fitness [90]. Another example also occurs in the larval stage, specifically in masked bird caterpillars (*Drepana arcuata*). During the first and second instars, caterpillars aggregate to build a common silk shelter on the leaf that they reside on, and feed in a coordinated manner [91]. Cooperative behaviour among larvae in natural populations of insects may be an important adaptation, due to the nature of this life stage involving the need for food and growth with limited dispersal ability. Further studies into larval social behaviour in other insect species may contribute more towards our knowledge of the evolution of cooperation in general.

### 3.3. Stress responses and physiology

Crowding as a stressor must be considered in an evolutionary context in terms of the different adaptations that each species evolves to overcome this stressor. Different species of *Drosophila* evolved different physiological adaptations to larval crowding. Previously we discussed *D. melanogaster* behavioural responses to crowding, such as increased feeding rate. *D. ananassae* and *D. n. nasuta* evolved other competitive abilities in response to larval crowding, such as an increased rate of nutrient conversion to biomass and a shortened developmental time [92]. The negative effects of crowding on fitness also differ by species. Under crowded conditions, *D. melanogaster* larvae have reduced larval viability, while *D. simulans* have lower fecundity [93]. Species-specific adaptation to crowding may reflect the fact that natural populations of *Drosophila* often deal with interspecific competition [62] and may have evolved to occupy different niches to maximize their competitive ability. While *D. melanogaster* and *D. simulans* coexist throughout most temperate and tropical habitats, studies have shown that even small differences in temperature, moisture, and food composition can change the relative fitness of one species compared to the other (reviewed in [94]). Differing competitive abilities and resource allocation (larval viability vs. adult fecundity) under crowding may be a mechanism to allow different species of *Drosophila* to thrive in specific environments.

Research on *D. melanogaster* larval crowding has shown many additional fitness-related effects that extend to the adult phase such as body size, lifespan, and stress resistance. Klepsatel et al. 2018 concluded that the limited availability of dietary yeast during the larval period was the main factor contributing to these effects [95]. Crowding increases lifespan [96] and thermotolerance [97], by inducing Hsp70 expression, a stress response that researchers characterized as having a ‘hardening/acclimation’ effect on the flies [98]. Another study found that supplementing the diet of flies with blueberry anthocyanins rescue some of the detrimental effects of crowding and reduced Hsp70 expression [99], providing more insights into how natural compounds aid in reversing oxidative stress. Heat-shock proteins are highly conserved in animals and are an important area of study due to their involvement in longevity and ageing [100]. While mechanisms of the heat-shock response have been studied extensively in *Drosophila* [101,102] future research specifically on the role of the social environment may further increase our understanding of the pathways that animals use to respond to stress.

## 4. Part 3: composition of the social environment

So far, we have focused mainly on *Drosophila’s* ability to sense and respond to differences in the presence and quantity of other individuals in their surroundings. However, natural populations are not homogenous. An individual may interact with many members of its species that vary in their genetics, influencing the types of social interactions in that encounter. When genes expressed in one individual affect the phenotype of another individual, it is known as an indirect genetic effect [IGE; reviewed in [103,104]]. Additionally, prior experiences of individuals may also influence their behaviour and shape the social environment (such as the outcome of a past aggressive interaction influencing the behaviour and the outcome of later aggressive encounters) [105]. Finally, in the case of *D. melanogaster*, many other species of *Drosophila* will often be present at a singular food source [106], providing opportunities for interspecific interactions.

The previous section touched on behavioural plasticity in mating based on recognition of the sex of surrounding individuals, and plasticity in cooperation based on kin recognition. This section explores this topic from the perspective of how flies gain information about the composition of their social environment and adjust their behaviour accordingly.

### 4.1. Mating and aggression

As previously discussed, crowding influences male courtship and mating behaviour through the increased perception of sperm competition. Females are also able to alter their mating behaviour based on their perception of group composition. Billeter et al. 2012 found that female *Drosophila* mate more frequently in heterogeneous groups containing males from multiple strains, which increases the genetic diversity of their offspring [107]. Analysis of sensory-impaired mutants revealed that olfaction in females is required for this change in mating frequency, indicating that the results are due to plasticity in female behaviour rather than male-male interactions. Much like how the perception of competition influences male mating behaviour, the perception of mate quality (via genetic diversity) influences female mating behaviour. The ability to alter the amount of energy invested in mating is an important evolutionary trait to maximize reproductive fitness.

Aggression over resource competition is also plastic and is dependent on both past experiences and group composition [108]. In this experiment, researchers manipulated populations of male *Drosophila* by mixing various ratios of fly strains that differed in aggression. Aggression was measured in flies before and after imposing a period of resource limitation on each strain. They found that group (strain) composition determined the direction of behavioural plasticity in response to resource availability. All males in homogenous groups increased aggressiveness, while in mixed groups only strains that were in the minority increased aggressiveness. This study is the first to show that aggressive behaviours do not only depend on the genotype of the individual and their current environment, but their past experiences as well. Social influences on human aggressive and violent behaviours are well studied [109]. The ability for a simple model organism to also show behavioural plasticity based on past social experiences may complement our understanding of the evolution of aggression.

### 4.2. Sensory perception, social networks, and group behaviour

Research has shown that chemical communication is a key component of many group interactions and behaviour in *Drosophila*. Schneider et al. 2012 first characterized social network parameters in flies by tracking the movements of flies within an arena [110]. They discovered that flies form non-random social networks, with interactions between individuals being structured rather than stochastic. These interactions are dependent on chemosensory cues, as groups of gustatory and olfactory mutants have disrupted social networks. Prior social experience increases the variability of social interactions within a network [111]. Genetics may also play a role, as different strains of flies form social networks with different properties [110]. The *for* gene is one example of a genetic influence on social network properties: rover and sitter strains differ in the duration and frequency of pairwise interactions between flies within social networks [112]. Pairwise interactions between rovers were shorter in duration but higher in frequency [112]. Social network parameters such as global efficiency (higher in rovers) and assortativity (higher in sitters) also differed between strains [112].

Another study investigating the evolution of social network properties in *Drosophila* found that behavioural elements of social networks (movement, social spacing, and pairwise interactions) are largely shaped by historical environmental factors such as climate [113]. Social networks serve many purposes in many insect groups, including disease transmission, foraging, mating, and oviposition [114]. Understanding social behaviour and the mechanisms of social structure in *Drosophila* may contribute to research on the evolution of more complex social organizations found in nature, such as in eusocial insects [115].

Chemical communication is also necessary for behaviours such as mating [67,68]. Researchers investigated the characteristics of pheromonal communication in males by extracting chemicals produced by groups of males [116]. It was found that the pheromonal profiles produced by males is influenced by the individual’s genotype, prior social experience, as well as the genotype of surrounding flies, which interact with other environmental factors such as the light/dark cycle [116]. The circadian rhythm of flies is also dependent on chemical signalling and can be altered by social influence [117]. Olfactory mutants are not able to synchronize their internal clocks compared to groups of wild-type flies [117].

Mechanosensation is also an important form of communication in groups of *Drosophila*, most notably for its role in collective behaviour. Collective behaviours in groups of animals are important in avoiding dangers such as predation [118,119], demonstrated that flies respond to an aversive odour faster in groups than individually. They can even communicate this to mutant flies that cannot sense the odour. This behaviour involves the no mechanoreceptor potential C (NOMPC) channels on distal leg mechanosensory neurons [119].

The role of vision as a form of communication and its role in social behaviour may be an emergent field of study in the future. Using machine learning and neural networks, Schneider et al. [120], showed that *Drosophila* are visually distinct, and that the fly visual system is capable in theory of distinguishing between individuals.

The research discussed in this section contributes to our understanding of the sensory systems and circuits in *Drosophila*, and provides a basis for studying how mechanisms of recognition and communication between individuals of a species influence social behaviour. This can be relevant in understanding how conserved sensory mechanisms evolved and what purpose they serve across species that vary in social behaviour.

### 4.3. Learning and the social environment

Many assays exist to investigate learning and memory in flies, such as the courtship conditioning assay [24] or olfactory shock avoidance classical conditioning learning [121,122]. More recently, flies have demonstrated the ability to learn from other individuals (known as social or group learning) [123,124]. One such example is mate copying: when given the choice to mate, females normally prefer mating with wild-type flies over *CyO* mutants. However, after observing another female fly copulating with a *CyO* male, a female is more likely to choose a *CyO* mate instead [125]. A possible consequence of this phenomenon is that genetic variation and the maintenance of fitness-reducing alleles in populations can be affected by social information. Danchin et al. 2018 also used social learning of mate choice in *D. melanogaster* flies to model the ability of groups of flies to form and pass on cultural traditions across generations [126]. This exciting study revealed that conformist social learning predicted mate-choice traditions in *D. melanogaster.*

The ability to engage in group learning may depend on the genotypes present. The *for* gene allelic variants (rover and sitter) are naturally polymorphic in their short and long-term memory abilities [127,128]. However, whether flies are trained/tested in the olfactory shock learning assay as individuals or in groups influences their performance: sitters learn more effectively in groups, but rovers perform similarly under either condition [129]. These studies show that there is natural genetic variation in individual learning, and that there can be gene by social environment interactions. The genetic composition of the social environment may also have an effect. A study examining the aggregation behaviour of rovers and sitters found that when navigating a heated arena, sitters were more likely to aggregate on cooler refuges that already had other flies present [86]. In comparison, rovers were more likely to distribute themselves evenly among refugees. When testing mixed-strain groups, rovers tended to increase their aggregation when they were in the minority, and sitters decreased their aggregation when they were in the minority, suggesting that each individual’s cognition and decision-making in navigating the arena depended on the behaviour of the surrounding flies. It remains to be seen how heterogenous populations containing both sitters and rovers can factor into an individual’s associative learning phenotype. The relative fitness of rover and sitter alleles is also dependent on their frequency within a population: if rovers are more rare, they have higher survivorship to adulthood, and vice-versa for sitters [130]. Future studies could investigate whether frequency-dependent selection also extends to the adult life stage and if it is connected to social behaviour.

Plasticity in learning based on the social environment also extends to interactions between different species of *Drosophila*. Flies use visual cues such as wing movements to communicate to other individuals about the presence of parasitoid wasps, who can then alter their behaviour by shifting food preferences and oviposition frequency [131]. Communication is more effective among members of the same species. However, through studying groups of *D. melanogaster* and *D. ananassae* that after living among another species for some time, inter-specific communication can be improved to be more efficient by learning the ‘dialects’ that the other species uses to communicate [131]. Communities consisting of multiple species of *Drosophila* may interact with each other in similar ways that individuals interact with members of their own species. From this perspective, research into the social behaviour of *Drosophila* species could be expanded to include interspecific interactions as well.

## 5. Conclusions

In this review, we have discussed experimental findings that address how aspects of the social environment such as isolation, crowding, and the composition of surrounding individuals affect *Drosophila melanogaster*. We have shown that *Drosophila* respond to social conditions in complex and multifactorial ways. Flies demonstrate a wide range of behavioural plasticity based on both perceptions of current social contexts, as well as past social experiences. Behaviour is also dependent on the age, sex, and genetics of the individual itself.

This field of research is crucial to providing a complete picture of the ecology and evolution of *Drosophila*. It provides a greater understanding of the challenges that these organisms face in nature in terms of their dynamic social environment, and the traits they have evolved to respond to them.

These studies also demonstrate that a model organism typically considered to be a non-social insect actually has a rich social life and can be used to study how individuals respond to their social environments. Within the insect class, there exists a wide range of social behaviours. Many insects are solitary (spend their lives isolated, except when mating) [132], while the label of ‘social’ insects is usually reserved for taxa within the Hymenoptera in which obligate eusociality evolved (characterized by the reproductive division of labour) [133]. *Drosophila* may be an excellent candidate to test sociobiological hypotheses for genes that mediate eusocial behaviour. Homologs of the *Drosophila for* gene are known to regulate the division of labour in bees and ants [reviewed in] [134]. *Drosophila* can also respond to the honeybee queen mandibular pheromone (released by honeybee queens to sterilize female workers in the colony), showing reduction in ovary development and fecundity [135]. We can also expect that future research in *Drosophila* can complement our understanding of mammalian and human social behaviour and health from the perspective of genetics and neurobiology, by analysis of conserved genes and pathways between species.

A final important takeaway from these studies is that future laboratory research utilizing *Drosophila* as a model organism must carefully consider the social environment as an important factor to control for in experimental methodology. Isolation, crowding, and other social parameters do not only affect behaviour, but also development, gene expression, and physiology. Additionally, effects can be observed across a lifetime or even across generations. Therefore, the social conditions that *Drosophila* are reared at across different stages of life must be recorded and controlled – as experiments may not be reproducible otherwise.

Acknowledgements: Research was supported by an NSERC Discovery Grant to MBS and an NSERC graduate fellowship to MC. Thanks to Sydney Gram and the FLY reviewers who provided helpful comments on our manuscript. This paper is dedicated to our dear friend and colleague Jean-Marc Jallon (1945–2019) whose fundamental research identified the sex pheromones of Drosophila and their functions in sexual communication. This work, elaborated by his students, defines a lexicon for communication, recognition and identity among flies.

## References

[1] Moore AJ, Brodie ED III, Wolf JB, et al. Interacting phenotypes and the evolutionary process: i. direct and indirect genetic effects of social interactions. Evolution. 1997;51(5):1352–1362. 28568644 10.1111/j.1558-5646.1997.tb01458.x

[2] Bekoff M. The development of social interaction, play, and metacommunication in mammals: an ethological perspective. Q Rev Biol. 1972;47(4):412–434.

[3] Creel S, Dantzer B, Goymann W, et al. The ecology of stress: effects of the social environment. Funct Ecol. 2013;27(1):66–80.

[4] Becker GS, Murphy KM. Social economics: market behavior in a social environment. Harvard University Press; 2009, Cambridge Mass.

[5] Yen IH, Syme SL. The social environment and health: a discussion of the epidemiologic literature. Annu Rev Public Health. 1999;20(1):287–308. 10352860 10.1146/annurev.publhealth.20.1.287

[6] Brookes, M. Fly: The Unsung Hero of 20th-Century Science. New York, NY: HarperCollins Publishers Inc; 2009.

[7] Reaume CJ, Sokolowski MB. The nature of *Drosophila melanogaster*. Curr Biol. 2006;16(16):R623–R628. 16920605 10.1016/j.cub.2006.07.042

[8] Sokolowski MB. *Drosophila*: genetics meets behavior. Nat Rev Genet. 2001;2(11):879–890. 11715043 10.1038/35098592

[9] Ferreira CH, Moita MA. What can a non-eusocial insect tell us about the neural basis of group behavior? Curr Opin Insect Sci. 2019;36:118–124. 31563022 10.1016/j.cois.2019.09.001

[10] Ramdya P, Schneider J, Levine JD, et al. The neurogenetics of group behavior in *Drosophila melanogaster*. J Exp Biol. 2017;220(1):35–41. 28057826 10.1242/jeb.141457

[11] Chappell NL, Badger M. Social isolation and well-being. J Gerontol. 1989;44(5):S169–S176. 2768776 10.1093/geronj/44.5.s169

[12] Nicholson Jr. NR Jr. Social isolation in older adults: an evolutionary concept analysis. J Adv Nurs. 2009;65(6):1342–1352. 19291185 10.1111/j.1365-2648.2008.04959.x

[13] Arakawa H. Ethological approach to social isolation effects in behavioral studies of laboratory rodents. Behav Brain Res. 2018;341:98–108. 29287909 10.1016/j.bbr.2017.12.022

[14] Einon DF, Morgan MJ. A critical period for social isolation in the rat. Dev Psychobiol. 1977;10(2):123–132. 838157 10.1002/dev.420100205

[15] Fone KC, Porkess MV. Behavioral and neurochemical effects of post-weaning social isolation in rodents-relevance to developmental neuropsychiatric disorders. Neurosci Biobehav Rev. 2008;32(6):1087–1102. 18423591 10.1016/j.neubiorev.2008.03.003

[16] Harlow HF, Dodsworth RO, Harlow MK, et al. Total social isolation in monkeys. Proc Nat Acad Sci. 1965;54(1):90–97. 4955132 10.1073/pnas.54.1.90 PMC285801

[17] Bailey NW, Moore AJ. Evolutionary consequences of social isolation. Trends in Ecology and & Evolution. 2018;33(8):595–607. 10.1016/j.tree.2018.05.008 30055910

[18] Pereira HS, Williams KD, De Belle JS, et al. Marla Sokolowski: and now for someone completely different. J Neurogenet. 2021. DOI:10.1080/01677063.2021.1940175. 34256677

[19] Judge KA. Female social experience affects the shape of sexual selection on males. Evol Ecol Res. 2010;12(3):389–402.

[20] Jarrett BJ, Schrader M, Rebar D, et al. Cooperative interactions within the family enhance the capacity for evolutionary change in body size. Nat Ecol Evol. 2017;1(7):1–7. 28685165 10.1038/s41559-017-0178 PMC5495167

[21] de Melo Sene F. Effect of social isolation on behavior of *Drosophila silvestris* from Hawaii. Proc. Hawaiian Ent Soc. 1977;22(3):469–474.

[22] Kim YK, Ehrman L. Developmental isolation and subsequent adult behavior of *Drosophila paulistorum*. IV. Courtship. Behav Genet. 1998;28(1):57–65. 9573647 10.1023/a:1021460832378

[23] Dukas R. Experience improves courtship in male fruit flies. An Behav. 2005;69(5):1203–1209.

[24] Koemans TS, Oppitz C, Donders RA, et al. *Drosophila* courtship conditioning as a measure of learning and memory. J Vis Exp. 2017;124:e55808. 10.3791/55808 PMC5608251 28605393

[25] Villella A, Hall JC. Neurogenetics of courtship and mating in *Drosophila*. Adv Genet. 2008;62:67–184. 19010254 10.1016/S0065-2660(08)00603-2

[26] von Schilcher F. A mutation which changes courtship song in *Drosophila melanogaster*. Behav Genet. 1977;7(3):251–259. 405965 10.1007/BF01066278

[27] Marie-Orleach L, Bailey NW, Ritchie MG, et al. Social effects on fruit fly courtship song. Ecol Evol. 2019;9 1140(1):410–416. 10.1002/ece3.4759 PMC6342107 30680123

[28] Sethi S, Lin HH, Shepherd AK, et al. Social context enhances hormonal modulation of pheromone detection in *Drosophila*. Curr Biol. 2019;29(22):3887–3898. 31679932 10.1016/j.cub.2019.09.045 PMC6911769

[29] Wijesekera TP, Saurabh S, Dauwalder B, et al. Juvenile hormone is required in adult males for *Drosophila* courtship. PloS One. 2016;11(3):e0151912. 27003411 10.1371/journal.pone.0151912 PMC4803231

[30] Bailey NW, Hoskins JL, Green J, et al. Measuring same-sex sexual behavior: the influence of the male social environment. An Behav. 2013;86(1):91–100.

[31] McRobert SP, Tompkins L. Two consequences of homosexual courtship performed by *Drosophila melanogaster* and *Drosophila affinis* males. Evolution. 1988;42(5):1093–1097. 28581176 10.1111/j.1558-5646.1988.tb02528.x

[32] Filice DC, Long TA. Phenotypic plasticity in female mate choice behavior is mediated by an interaction of direct and indirect genetic effects in *Drosophila melanogaster*. Ecol Evol. 2017;7(10):3542–3551. 28515889 10.1002/ece3.2954 PMC5433979

[33] Slepian Z, Sundby K, Glier S, et al. Visual attraction in *Drosophila* larvae develops during a critical period and is modulated by crowding conditions. J Comp Physiol A. 2015;201(10):1019–1027. 10.1007/s00359-015-1034-3 26265464

[34] Nijhout HF. The control of body size in insects. Dev Biol. 2003;261(1):1–9. 12941617 10.1016/s0012-1606(03)00276-8

[35] Edelsparre AH, Vesterberg A, Lim JH, et al. Alleles underlying larval foraging behavior influence adult dispersal in nature. Ecol Lett. 2014;17(3):333–339. 24386971 10.1111/ele.12234

[36] Leech T, Sait SM, Bretman A, et al. Sex-specific effects of social isolation on ageing in *Drosophila melanogaster*. J Insect Physiol. 2017;102:12–17. 28830760 10.1016/j.jinsphys.2017.08.008

[37] Leech T, Evison SE, Armitage SA, et al. Interactive effects of social environment, age and sex on immune responses in *Drosophila melanogaster*. J Evol Biol. 2019;32(10):1082–1092. 31313398 10.1111/jeb.13509

[38] Burns JG, Svetec N, Rowe L, et al. Gene–environment interplay in *Drosophila melanogaster*: chronic food deprivation in early life affects adult exploratory and fitness traits. Proc Nat Acad Sci. 2012;109(Supplement 2), 17239–17244. 23045644 10.1073/pnas.1121265109 PMC3477394

[39] Wauben IP, Wainwright PE. The influence of neonatal nutrition on behavioral development: a critical appraisal. Nutr Rev. 1999;57(2):35–44. 10079701 10.1111/j.1753-4887.1999.tb01776.x

[40] Scrimshaw NS. Malnutrition, brain development, learning, and behavior. Nutr Res. 1998;18(2):351–379.

[41] Chi MW, Griffith LC, Vecsey CG, et al. Larval population density alters adult sleep in wild-type *Drosophila melanogaster* but not in amnesiac mutant flies. Brain Sci. 2014;4(3):453–470. 25116571 10.3390/brainsci4030453 PMC4194033

[42] Everson CA. Physiological consequences of sleep deprivation. J Musculoskelet Pain. 1998;6(3):93–101.

[43] Beattie L, Kyle SD, Espie CA, et al. Social interactions, emotion and sleep: a systematic review and research agenda. Sleep Med Rev. 2015;24:83–100. 25697832 10.1016/j.smrv.2014.12.005

[44] Kaushal N, Nair D, Gozal D, et al. Socially isolated mice exhibit a blunted homeostatic sleep response to acute sleep deprivation compared to socially paired mice. Brain Res. 2012;1454:65–79. 22498175 10.1016/j.brainres.2012.03.019 PMC3662550

[45] Yu B, Steptoe A, Niu K, et al. Prospective associations of social isolation and loneliness with poor sleep quality in older adults. Qual Life Res. 2018;27(3):683–691. 29188485 10.1007/s11136-017-1752-9

[46] Ganguly-Fitzgerald I, Donlea J, Shaw PJ, et al. Waking experience affects sleep need in *Drosophila*. Science. 2006;313(5794):1775–1781. 16990546 10.1126/science.1130408

[47] Brown MK, Strus E, Naidoo N, et al. Reduced sleep during social isolation leads to cellular stress and induction of the unfolded protein response. Sleep. 2017;40(7)zsz095. 10.1093/sleep/zsx095 PMC6075573 28541519

[48] Li W, Wang Z, Syed S, et al. Chronic social isolation signals starvation and reduces sleep in *Drosophila*. Nature. 2021;597(7875):239–244. 34408325 10.1038/s41586-021-03837-0 PMC8429171

[49] Liu G, Nath T, Linneweber GA, et al. A simple computer vision pipeline reveals the effects of isolation on social interaction dynamics in *Drosophila*. PLoS Comput Biol. 2018;14(8):e1006410. 30161262 10.1371/journal.pcbi.1006410 PMC6135522

[50] Agrawal P, Chung P, Heberlein U, et al. Enabling cell-type-specific behavioral epigenetics in *Drosophila*: a modified high-yield INTACT method reveals the impact of social environment on the epigenetic landscape in dopaminergic neurons. BMC Biol. 2019;17(1):1–19. 30967153 10.1186/s12915-019-0646-4 PMC6456965

[51] Simon AF, Chou MT, Salazar ED, et al. A simple assay to study social behavior in *Drosophila*: measurement of social space within a group. Genes Brain Behav. 2012;11(2):243–252. 22010812 10.1111/j.1601-183X.2011.00740.x PMC3268943

[52] Fernandez RW, Akinleye AA, Nurilov M, et al. Modulation of social space by dopamine in *Drosophila melanogaster*, but no effect on the avoidance of the *Drosophila* stress odorant. Biol Lett. 2017;13(8):20170369. 28794277 10.1098/rsbl.2017.0369 PMC5582115

[53] Ueda A, Kidokoro Y. Aggressive behaviors of female *Drosophila melanogaster* are influenced by their social experience and food resources. Physiol Entomol. 2002;27(1):21–28.

[54] Hoffmann AA. The influence of age and experience with conspecifics on territorial behavior in *Drosophila melanogaster*. J. Insect Behav. 1990;27:1–12.

[55] Belenioti M, Chaniotakis N. Aggressive behavior of *Drosophila suzukii* in relation to environmental and social factors. Sci Rep. 2020;10(1):1–10. 32398716 10.1038/s41598-020-64941-1 PMC7217943

[56] Markow TA. The natural history of model organisms: the secret lives of *Drosophila* flies. Elife. 2015;4:e06793. 26041333 10.7554/eLife.06793 PMC4454838

[57] Ueda A, Wu CF. Effects of social isolation on neuromuscular excitability and aggressive behaviors in *Drosophila*: altered responses by Hk and gsts1, two mutations implicated in redox regulation. J Neuro. 2009;23(4):378–394. 10.3109/01677060903063026 PMC3632667 19863269

[58] Agrawal P, Kao D, Chung P, et al. The neuropeptide drosulfakinin regulates social isolation-induced aggression in *Drosophila*. J Exp Biol. 2020;223(2):jeb207407. 10.1242/jeb.207407 PMC7033730 31900346

[59] Skibicka KP, Dickson SL. Enteroendocrine hormones—central effects on behavior. Curr Opin Pharmacol. 2013;13(6):977–982. 24091195 10.1016/j.coph.2013.09.004

[60] Lenka A, Arumugham SS, Christopher R, et al. Genetic substrates of psychosis in patients with parkinson’s disease: a critical review. J Neurol Sci. 2016;364:33–41. 27084212 10.1016/j.jns.2016.03.005

[61] Rohde PD, Gaertner B, Ward K, et al. Genomic analysis of genotype-by-social environment interaction for *Drosophila melanogaster* aggressive behavior. Genetics. 2017;206(4):1969–1984. 28550016 10.1534/genetics.117.200642 PMC5560801

[62] Atkinson WD. A field investigation of larval competition in domestic *Drosophila*. J Anim Ecol. 1979;91–102.

[63] Loo C. Important issues in researching the effects of crowding on humans. Crowding and Behavior. New York, NY: Arno Press;1974.

[64] D’Atri DA. Psychophysiological responses to crowding. Environment and Behavior. 1975;7(2):237–252.

[65] Rooke R, Rasool A, Schneider J, et al. *Drosophila melanogaster* behavior changes in different social environments based on group size and density. Commun Biol. 2020;3(1):1–6. 32533063 10.1038/s42003-020-1024-z PMC7293324

[66] Billeter J, Levine JD. The role of cVA and the odorant binding protein lush in social and sexual behavior in *Drosophila melanogaster*. Front Ecol and Evol. 2015;3:75.

[67] Ferveur JF. Cuticular hydrocarbons: their evolution and roles in *Drosophila* pheromonal communication. Behav Genet. 2005;35(3):279–295. 15864443 10.1007/s10519-005-3220-5

[68] Jallon JM. A few chemical words exchanged by *Drosophila* during courtship and mating. Behav Genet. 1984;14(5):441–478. 6441563 10.1007/BF01065444

[69] Crossley S, Wallace B. The effects of crowding on courtship and mating success in *Drosophila melanogaster*. Behav Genet. 1987;17(5):513–522. 3122719 10.1007/BF01073118

[70] Shenoi VN, Banerjee SM, Guruswamy B, et al. *Drosophila melanogaster* males evolve increased courtship as a correlated response to larval crowding. An Behav. 2016;120:183–193.

[71] Dasgupta P, Sarkar S, Das AA, et al. Intergenerational paternal effect of adult density in *Drosophila melanogaster*. Ecol Evol. 2019;9(6):3553–3563. 30962910 10.1002/ece3.4988 PMC6434557

[72] Omesi L, Levi M, Bentzur A, et al. Sexual deprivation modulates social interaction and reproductive physiology. bioRxiv; 2021.

[73] Shohat-Ophir G, Kaun KR, Azanchi R, et al. Sexual deprivation increases ethanol intake in *Drosophila*. Science. 2012;335(6074):1351–1355. 22422983 10.1126/science.1215932 PMC3909676

[74] Edelsparre AH, Fitzpatrick MJ, Rodriguez MA, et al. Tracking dispersal across a patchy landscape reveals a dynamic interaction between genotype and habitat structure. Oikos. 2021;130(1):79–94.

[75] Lakovic M, Poethke HJ, Hovestadt T, et al. Dispersal timing: emigration of insects living in patchy environments. PLoS One. 2015;10(7):e0128672. 26132493 10.1371/journal.pone.0128672 PMC4489195

[76] Lambin X, Aars, J, Piertney, SB. Dispersal, intraspecific competition, kin competition, and kin facilitation: A review of the empirical evidence. In J. Clobert, E. Danchin, A. A. Dhondt, & J. D. Nichols (Editors)., Dispersal Oxford University Press. 2001.

[77] Matthysen E. Density-dependent dispersal in birds and mammals. Ecography. 2005;28(3):403–416.

[78] Guo PZ, Mueller LD, Ayala FJ, et al. Evolution of behavior by density-dependent natural selection. Proc Nat Acad Sci. 1991;88(23):10905–10906. 1961760 10.1073/pnas.88.23.10905 PMC53040

[79] Joshi A, Mueller LD. Evolution of higher feeding rate in *Drosophila* due to density-dependent natural selection. Evolution. 1988;42(5):1090–1093. 28581181 10.1111/j.1558-5646.1988.tb02527.x

[80] Sokolowski MB. Foraging strategies of *Drosophila melanogaster*: a chromosomal analysis. Behav Genet. 1980;10(3):291–302. 6783027 10.1007/BF01067774

[81] Osborne KA, Robichon A, Burgess E, et al. Natural behavior polymorphism due to a cGMP-dependent protein kinase of *Drosophila*. Science. 1997;277(5327):834–836. 9242616 10.1126/science.277.5327.834

[82] Sokolowski MB, Pereira HS, Hughes K, et al. Evolution of foraging behavior in *Drosophila* by density-dependent selection. Proc Nat Acad Sci. 1997;94(14):7373–7377. 9207098 10.1073/pnas.94.14.7373 PMC23828

[83] Durisko Z, Dukas R. Attraction to and learning from social cues in fruit fly larvae. Proc R Soc B. 2013;280(1767):20131398. 10.1098/rspb.2013.1398 PMC3735258 23902906

[84] Rohlfs M. Clash of kingdoms or why *Drosophila* larvae positively respond to fungal competitors. Front Zool. 2005;2(1):1–7. 15679898 10.1186/1742-9994-2-2 PMC548382

[85] Trienens M, Rohlfs M. A potential collective defense of *Drosophila* larvae against the invasion of a harmful fungus. Front Ecol and Evol. 2020;8:1–10.

[86] Philippe AS, Jeanson R, Pasquaretta C, et al. Genetic variation in aggregation behavior and interacting phenotypes in *Drosophila*. Proc R Soc B. 2016;283(1827):20152967. 10.1098/rspb.2015.2967 PMC4822458 27009219

[87] Dombrovski M, Poussard L, Moalem K, et al. Cooperative behavior emerges among *Drosophila* larvae. Curr Biol. 2017;27(18):2821–2826. 28918946 10.1016/j.cub.2017.07.054

[88] Khodaei L, Long TA. Kin recognition and co-operative foraging in *Drosophila melanogaster* larvae. J Evol Biol. 2019;32(12):1352–1361. 31454451 10.1111/jeb.13531

[89] Dombrovski M, Kuhar R, Mitchell A, et al. Cooperative foraging during larval stage affects fitness in *Drosophila*. J Comp Physiol A. 2020;206(5):743–755. 10.1007/s00359-020-01434-6 PMC7392940 32623493

[90] Rebar D, Bailey NW, Jarrett BJ, et al. An evolutionary switch from sibling rivalry to sibling cooperation, caused by a sustained loss of parental care. Proc Nat Acad Sci. 2020;117(5):2544–2550. 31964847 10.1073/pnas.1911677117 PMC7007579

[91] Yadav C, Smith ML, Yack JE, et al. Transcriptome analysis of a social caterpillar, *Drepana arcuata*: De novo assembly, functional annotation and developmental analysis. PloS One. 2020;15(6):e0234903. 32569288 10.1371/journal.pone.0234903 PMC7307738

[92] Sarangi M, Nagarajan A, Dey S, et al. Evolution of increased larval competitive ability in *Drosophila melanogaster* without increased larval feeding rate. J Genet. 2016;95(3):491–503. 27659320 10.1007/s12041-016-0656-8

[93] Barker JSF. Adult population density, fecundity and productivity in *Drosophila melanogaster* and *Drosophila simulans*. Oecologia. 1973;11(2):83–92. 28307208 10.1007/BF00345125

[94] Miller RS. Larval competition in *Drosophila melanogaster* and *D. simulans*. Ecology. 1964;45(1):132–148.

[95] Klepsatel P, Procházka E, Gáliková M, et al. Crowding of *Drosophila* larvae affects lifespan and other life-history traits via reduced availability of dietary yeast. Exp Gerontol. 2018;110:298–308. 29932967 10.1016/j.exger.2018.06.016

[96] Miller RS, Thomas, JL. The effects of larval crowding and body size on the longevity of adult *Drosophila melanogaster*. Ecology. 1958;39(1):118–125.

[97] Henry Y, Renault D, Colinet H, et al. Hormesis-like effect of mild larval crowding on thermotolerance in *Drosophila* flies. J Exp Biol. 2018;221(2);jeb.178681. 10.1242/jeb.169342 29191860

[98] Sørensen JG, Loeschcke V. Larval crowding in *Drosophila melanogaster* induces Hsp70 expression, and leads to increased adult longevity and adult thermal stress resistance. J Insect Physiol. 2001;47(11):1301–1307. 12770182 10.1016/s0022-1910(01)00119-6

[99] Zhang Z, Tang C, Huang X, et al. Blueberry anthocyanins effectively relieve the stress of population crowding in *Drosophila melanogaster*. Chinese J. App. Ent. 2016;53(2): 331–339

[100] Calderwood SK, Murshid A, Prince T, et al. The shock of aging: molecular chaperones and the heat shock response in longevity and aging–a mini-review. Gerontology. 2009;55(5):550–558. 19546513 10.1159/000225957 PMC2754743

[101] Lindquist S, Craig EA. The heat-shock proteins. Annu Rev Genet. 1988;22(1):631–677. 2853609 10.1146/annurev.ge.22.120188.003215

[102] Pauli D, Arrigo AP, Tissières A, et al. Heat shock response in *Drosophila*. Experientia. 1992;48(7):623–629. 1639169 10.1007/BF02118306

[103] Bailey NW, Marie-Orleach L, Moore AJ. Indirect genetic effects in behavioral ecology: does behavior play a special role in evolution? Behav Ecology. 2018;29(1):1–11.

[104] Wolf JB, Brodie ED III, Cheverud JM, et al. Evolutionary consequences of indirect genetic effects. Trends Ecol Evol. 1998;13(2):64–69. 21238202 10.1016/s0169-5347(97)01233-0

[105] Hsu Y, Earley RL, Wolf LL, et al. Modulation of aggressive behavior by fighting experience: mechanisms and contest outcomes. Biol Rev. 2006;81(1):33–74. 16460581 10.1017/S146479310500686X

[106] Markow TA, O’Grady P. Reproductive ecology of *Drosophila*. Funct Ecol. 2008;22(5):747–759.

[107] Billeter JC, Jagadeesh S, Stepek N, et al. *Drosophila melanogaster* females change mating behavior and offspring production based on social context. Proc R Soc B. 2012;279(1737):2417–2425. 10.1098/rspb.2011.2676 PMC3350680 22298851

[108] Kilgour RJ, Norris DR, McAdam AG, et al. Carry-over effects of resource competition and social environment on aggression. Behav Ecol. 2020;31(1):140–151.

[109] Anderson CA, Bushman BJ. Human aggression. Annu Rev Psychol. 2002;53:27–51. 11752478 10.1146/annurev.psych.53.100901.135231

[110] Schneider J, Dickinson MH, Levine JD, et al. Social structures depend on innate determinants and chemosensory processing in *Drosophila*. Proc Nat Acad Sci. 2012;109(Supplement 2):17174–17179. 22802679 10.1073/pnas.1121252109 PMC3477376

[111] Bentzur A, Ben-Shaanan S, Benichou JI, et al. Early life experience shapes male behavior and social networks in *Drosophila*. Curr Biol. 2021;31(3):486–501. 33186552 10.1016/j.cub.2020.10.060

[112] Alwash N, Allen AM, Sokolowski B, et al. The *Drosophila melanogaster* foraging gene affects social networks. J Neurogenet. 2021:1–13. 10.1080/01677063.2021.1936517 34121597

[113] Jezovit JA, Rooke R, Schneider J, et al. Behavioral and environmental contributions to drosophilid social networks. Proc Nat Acad Sci. 2020;117(21): 11573–11583. 32404421 10.1073/pnas.1920642117 PMC7261129

[114] Alwash N, Levine JD. Network analyses reveal structure in insect social groups. Curr Opin Insect Sci. 2019;35:54–59. 31394418 10.1016/j.cois.2019.07.001

[115] Sokolowski MB. Social interactions in “simple” model systems. Neuron. 2010;65(6):780–794. 20346755 10.1016/j.neuron.2010.03.007

[116] Kent C, Azanchi R, Smith B, et al. Social context influences chemical communication in *D. melanogaster* males. Curr Biol. 2008;18(18):1384–1389. 18789689 10.1016/j.cub.2008.07.088

[117] Levine JD, Funes P, Dowse HB, et al. Resetting the circadian clock by social experience in *Drosophila melanogaster*. Science. 2002;298(5600):2010–2012. 12471264 10.1126/science.1076008

[118] Herbert-Read JE, Rosén E, Szorkovszky A, et al. How predation shapes the social interaction rules of shoaling fish. Proc R Soc B. 2017;284(1861):20171126. 10.1098/rspb.2017.1126 PMC5577484 28855361

[119] Ramdya P, Lichocki P, Cruchet S, et al. Mechanosensory interactions drive collective behavior in *Drosophila*. Nature. 2015;519(7542):233–236. 25533959 10.1038/nature14024 PMC4359906

[120] Schneider J, Murali N, Taylor GW, et al. Can *Drosophila melanogaster* tell who’s who? PloS One. 2018;13(10):e0205043. 30356241 10.1371/journal.pone.0205043 PMC6200205

[121] Malik BR, Hodge JJ. *Drosophila* adult olfactory shock learning. J Vis Exp. 2014;(90):e50107. 10.3791/50107 PMC4672959 25145496

[122] Tully T, Quinn WG. Classical conditioning and retention in normal and mutant *Drosophila melanogaster*. J Comp Physiol A. 1985;157(2):263–277. 3939242 10.1007/BF01350033

[123] Mery F, Varela SA, Danchin E, Blanchet S, Parejo D, Coolen I, Wagner RH. Public versus personal information for mate copying in an invertebrate. Curr. Biol. 2009;19(9):730–4. 10.1016/j.cub.2009.02.064 19361993

[124] Battesti M, Moreno C, Joly D, Mery F. Spread of social information and dynamics of social transmission within Drosophila groups. Curr. Biol. 2012;22:309–13. 10.1016/j.cub.2011.12.050 22264604

[125] Nöbel S, Danchin E, Isabel G, et al. Mate-copying for a costly variant in *Drosophila melanogaster* females. Behav Ecol. 2018;29(5):1150–1156.

[126] Danchin E, Nöbel S, Pocheville A, et al. Cultural flies: conformist social learning in fruit flies predicts long-lasting mate-choice traditions. Science. 2018;362(6418):1025–1030. 30498121 10.1126/science.aat1590

[127] Kaun KR, Hendel T, Gerber B, et al. Natural variation in *Drosophila* larval reward learning and memory due to a cGMP-dependent protein kinase. Learn Memory. 2007;14(5):342–349. 10.1101/lm.505807 PMC1876758 17522025

[128] Mery F, Belay AT, So AKC, et al. Natural polymorphism affecting learning and memory in *Drosophila*. Proc Nat Acad Sci. 2007;104(32):13051–13055. 17640898 10.1073/pnas.0702923104 PMC1941815

[129] Kohn NR, Reaume CJ, Moreno C, et al. Social environment influences performance in a cognitive task in natural variants of the foraging gene. PloS One. 2013;8(12):e81272. 24349049 10.1371/journal.pone.0081272 PMC3861308

[130] Fitzpatrick MJ, Feder E, Rowe L, et al. Maintaining a behavior polymorphism by frequency-dependent selection on a single gene. Nature. 2007;447(7141):210–212. 17495926 10.1038/nature05764

[131] Kacsoh BZ, Bozler J, Hodge S, et al. Neural circuitry of social learning in *Drosophila* requires multiple input to facilitate inter-species communication. Commun Biol. 2019;2(1):1–14. 31428697 10.1038/s42003-019-0557-5 PMC6692349

[132] Free J. Solitary and social insects. Nature. 1969;224 (5226):1336.

[133] Nowak MA, Tarnita CE, Wilson EO, et al. The evolution of eusociality. Nature. 2010;466(7310):1057–1062. 20740005 10.1038/nature09205 PMC3279739

[134] Lucas C, Ben-Shahar Y. The foraging gene as a modulator of division of labour in social insects. J Neurogenet. 2021. 35(3):168–178. 34151702 10.1080/01677063.2021.1940173

[135] Camiletti AL, Percival-Smith A, Thompson GJ, et al. Honey bee queen mandibular pheromone inhibits ovary development and fecundity in a fruit fly. Entomol Exp Appl. 2013;147(3):262–268.

